# Supplementary material for: Creating an atlas of normal tissue for pruning WSI patching through anomaly detection
Source: Sci Rep. 2024 Feb 16;14:3932. doi: 10.1038/s41598-024-54489-9 (PMC10873359; doi:10.1038/s41598-024-54489-9)
Supplement: Supplementary file 1 — Supplementary Tables. [file 41598_2024_54489_MOESM1_ESM.docx]

Supplementary Material

**Table S1.** Indexing and search F1 score leave-one-patient-out validation for top-1, top-3, and top-5 consensus predictions in skin dataset for each class, deep network, and experimental setup separately (DC = infiltrating ductal carcinoma, LC = lobular carcinoma, NL = normal).

| **Deep Network** | **Experiment Setup Top N** | **WD** | **MD** | **PD** | **NL** | **Weighted Average F1 Score** |
| --- | --- | --- | --- | --- | --- | --- |
|  | Top 1 | 0.86 | 0.35 | 0.58 | 0.96 | 0.77 |
|  | No Normal Atlas Top 3 | 0.88 | 0.35 | 0.67 | 0.96 | 0.79 |
|  | Top 5 | 0.89 | 0.32 | 0.67 | 0.97 | 0.80 |
|  | Top 1 | 0.84 | 0.34 | 0.59 | 0.93 | 0.75 |
| KimiaNet | Normal Atlas Top 3 | 0.88 | 0.38 | 0.62 | 0.92 | 0.78 |
|  | Top 5 | 0.89 | 0.42 | 0.70 | 0.94 | 0.81 |
|  | Top 1 | 0.81 | 0.38 | 0.59 | 0.76 | 0.71 |
|  | Normal Atlas Top 3 | 0.85 | 0.47 | 0.66 | 0.79 | 0.77 |
|  | Top 5 | 0.85 | 0.45 | 0.69 | 0.79 | 0.76 |
|  | Top 1 | 0.82 | 0.31 | 0.58 | 0.97 | 0.74 |
|  | No Normal Atlas Top 3 | 0.86 | 0.35 | 0.66 | 0.99 | 0.78 |
|  | Top 5 | 0.88 | 0.33 | 0.71 | 0.98 | 0.80 |
|  | Top 1 | 0.80 | 0.25 | 0.44 | 0.78 | 0.67 |
| ViT DINO | Normal Atlas Top 3 | 0.83 | 0.25 | 0.41 | 0.79 | 0.69 |
|  | Top 5 | 0.85 | 0.22 | 0.41 | 0.82 | 0.71 |
|  | Top 1 | 0.81 | 0.26 | 0.53 | 0.84 | 0.70 |
|  | Normal Atlas Top 3 | 0.84 | 0.27 | 0.61 | 0.86 | 0.73 |
|  | Top 5 | 0.85 | 0.30 | 0.62 | 0.88 | 0.75 |
|  | Top 1 | 0.83 | 0.30 | 0.48 | 0.95 | 0.73 |
|  | No Normal Atlas Top 3 | 0.84 | 0.18 | 0.50 | 0.95 | 0.72 |
|  | Top 5 | 0.87 | 0.20 | 0.61 | 0.95 | 0.75 |
|  | Top 1 | 0.50 | 0.26 | 0.37 | 0.41 | 0.43 |
| ResNet50 DINO | Normal Atlas Top 3 | 0.54 | 0.22 | 0.42 | 0.41 | 0.46 |
|  | Top 5 | 0.54 | 0.18 | 0.48 | 0.41 | 0.46 |
|  | Top 1 | 0.78 | 0.16 | 0.38 | 0.82 | 0.65 |
|  | Normal Atlas Top 3 | 0.83 | 0.17 | 0.36 | 0.83 | 0.68 |
|  | Top 5 | 0.84 | 0.16 | 0.36 | 0.82 | 0.69 |

via Isolation Forest

via One-Class SVM

via Isolation Forest

via One-Class SVM

via Isolation Forest

via One-Class SVM

**Table S2.** Indexing and search F1 score leave-one-patient-out validation for top-1, top-3, and top-5 consensus predictions in breast dataset for each class, deep network, and experimental setup separately (DC = infiltrating ductal carcinoma, LC = lobular carcinoma, NL = normal).

| **Deep Network** | **Experiment Setup Top N** | **DC** | **LC** | **NL** | **Weighted Average F1 Score** |
| --- | --- | --- | --- | --- | --- |
|  | Top 1 | 0.88 | 0.30 | 1.00 | 0.79 |
|  | No Normal Atlas Top 3 | 0.89 | 0.19 | 0.98 | 0.77 |
|  | Top 5 | 0.90 | 0.13 | 0.98 | 0.77 |
|  | Top 1 | 0.88 | 0.34 | 0.89 | 0.79 |
| KimiaNet | Normal Atlas Top 3 | 0.89 | 0.21 | 0.86 | 0.77 |
|  | Top 5 | 0.89 | 0.07 | 0.83 | 0.74 |
|  | Top 1 | 0.89 | 0.32 | 0.93 | 0.79 |
|  | Normal Atlas Top 3 | 0.89 | 0.18 | 0.81 | 0.76 |
|  | Top 5 | 0.89 | 0.13 | 0.81 | 0.76 |
|  | Top 1 | 0.88 | 0.39 | 1.00 | 0.80 |
|  | No Normal Atlas Top 3 | 0.89 | 0.24 | 1.00 | 0.78 |
|  | Top 5 | 0.90 | 0.17 | 1.00 | 0.78 |
|  | Top 1 | 0.88 | 0.37 | 0.73 | 0.78 |
| ViT DINO | Normal Atlas Top 3 | 0.89 | 0.29 | 0.69 | 0.77 |
|  | Top 5 | 0.89 | 0.09 | 0.69 | 0.74 |
|  | Top 1 | 0.87 | 0.37 | 0.55 | 0.77 |
|  | Normal Atlas Top 3 | 0.88 | 0.29 | 0.44 | 0.76 |
|  | Top 5 | 0.88 | 0.21 | 0.25 | 0.74 |
|  | Top 1 | 0.87 | 0.27 | 1.00 | 0.77 |
|  | No Normal Atlas Top 3 | 0.88 | 0.15 | 1.00 | 0.76 |
|  | Top 5 | 0.89 | 0.11 | 1.00 | 0.76 |
|  | Top 1 | 0.86 | 0.17 | 0.73 | 0.74 |
| ResNet50 DINO | Normal Atlas Top 3 | 0.88 | 0.15 | 0.60 | 0.74 |
|  | Top 5 | 0.88 | 0.11 | 0.32 | 0.72 |
|  | Top 1 | 0.88 | 0.31 | 0.89 | 0.78 |
|  | Normal Atlas Top 3 | 0.88 | 0.20 | 0.89 | 0.77 |
|  | Top 5 | 0.89 | 0.16 | 0.76 | 0.76 |

via Isolation Forest

via One-Class SVM

via Isolation Forest

via One-Class SVM

via Isolation Forest

via One-Class SVM

**Table S3.** Average number of patches, selection time, search time, and total time per WSI in three different experimental setups including no normal atlas, normal atlas with Isolation Forest, and normal atlas with one-class SVM for skin and breast WSIs. For each setup, the numbers have been reported for deep features obtained from all three networks. All times are reported in milliseconds.

| **Experiment Setup** | **Average per WSI** | **No Normal Atlas** | **Normal Atlas via Isolation Forest** | **Normal Atlas via One-Class SVM** |
| --- | --- | --- | --- | --- |
|  | Patches (number) | 66.4 | 33 | 21.6 |

|  | KimiaNet | Selection Time (ms)  Search Time (ms) | -  3112 | 28  2825 | 21  2481 |
| --- | --- | --- | --- | --- | --- |
|  |  | Total Time (ms) | 3112 | 2853 | 2502 |
|  |  | Patches (number) | 66.5 | 11.7 | 33.8 |
| Skin | ViT | Selection Time (ms) | - | 23 | 17 |
|  | DINO | Search Time (ms) | 2929 | 1466 | 3292 |
|  |  | Total Time (ms) | 2929 | 1489 | 3309 |
|  |  | Patches (number) | 66.4 | 13.4 | 9.4 |
|  | ResNet50 | Selection Time (ms) | - | 28 | 54 |
|  | DINO | Search Time (ms) | 3872 | 817 | 2379 |
|  |  | Total Time (ms) | 3872 | 845 | 2433 |
|  |  | Patches (number) | 248.5 | 216.5 | 199.1 |

|  | KimiaNet | Selection Time (ms)  Search Time (ms) | -  8957 | 75  6920 | 48  5792 |
| --- | --- | --- | --- | --- | --- |
|  |  | Total Time (ms) | 8957 | 6995 | 5840 |
|  |  | Patches (number) | 248.5 | 132.8 | 219.3 |
| Breast | ViT | Selection Time (ms) | - | 50 | 37 |
|  | DINO | Search Time (ms) | 6628 | 2413 | 5255 |
|  |  | Total Time (ms) | 6628 | 2463 | 5293 |
|  |  | Patches (number) | 248.4 | 144.9 | 134.6 |
|  | ResNet50 | Selection Time (ms) | - | 83 | 91 |
|  | DINO | Search Time (ms) | 17932 | 6307 | 5500 |
|  |  | Total Time (ms) | 17932 | 6390 | 5591 |
